# Sengstaken–Blakemore tube as bridge therapy to thoracic endovascular aortic repair for aortoesophageal fistula after heavy-ion radiotherapy for esophageal cancer

**DOI:** 10.1093/gastro/goag110

**Published:** 2026-10-24

**Authors:** Ryoma Sakamoto, Kenichiro Nakachi, Kimio Hosaka, Yuji Kato, Hiroshi Kusanagi

**Affiliations:** Department of Gastrointestinal Surgery, Kameda Medical Center, 929, Higashi-cho, Kamogawa, Chiba, 2960041, Japan; Department of Gastroenterology, Kameda Medical Center, 929, Higashi-cho, Kamogawa, Chiba, 2960041, Japan; Department of Cardiac and Vascular Surgery, Kameda Medical Center, 929, Higashi-cho, Kamogawa, Chiba, 2960041, Japan; Department of Cardiac and Vascular Surgery, Kameda Medical Center, 929, Higashi-cho, Kamogawa, Chiba, 2960041, Japan; Department of Gastrointestinal Surgery, Kameda Medical Center, 929, Higashi-cho, Kamogawa, Chiba, 2960041, Japan

## Introduction

Aortoesophageal fistula (AEF) is a rare and highly lethal condition. Although thoracic endovascular aortic repair (TEVAR) provides rapid hemostasis, emergency TEVAR may not be immediately available at all institutions. We describe a case in which a Sengstaken–Blakemore (SB) tube was used as a bridge to TEVAR.

## Case report

A 76-year-old man was brought to our hospital with a 3-day history of chest pain and hematemesis since the morning of presentation. He had received heavy-ion radiotherapy for esophageal cancer 7 months prior to presentation. Heavy-ion radiotherapy was delivered to the esophageal lesion in 12 fractions at a total dose of 50.4 Gy. He had also visited our hospital 2 months prior because of chest pain, and an esophagogastroduodenoscopy (EGD) performed at that time revealed a deep esophageal ulcer (Fig. 1A), which was considered a late complication of heavy-ion radiotherapy. Macroscopically, no residual tumor was identified. Although the patient was hemodynamically stable upon arrival, he subsequently developed transient loss of consciousness accompanied by hypotension. His hemoglobin level decreased from 9.2 to 6.7 g/dL, prompting blood transfusion. Computed tomography (CT) demonstrated communication between the gastroesophageal junction and the anterior aortic wall, along with a large gastric hematoma (Fig. 1B). Despite massive transfusion, the patient’s blood pressure remained unstable, necessitating urgent hemostasis with TEVAR. However, the cardiovascular surgery team was attending to another patient, precluding emergency TEVAR. Therefore, an 18-Fr SB tube was inserted transnasally. The gastric balloon was initially inflated with 100 mL of air. After its position was confirmed by gentle traction, an additional 100 mL of air was injected. The esophageal balloon was inflated with air to a pressure of 30 mm Hg using a pressure manometer. Immediately after placement, the systolic blood pressure stabilized and remained above 90 mm Hg. Following hemodynamic stabilization, the patient was transferred to the intensive care unit (ICU), with TEVAR scheduled for the following day. Although TEVAR should ideally be performed as soon as possible, a semi-elective approach was considered acceptable given the patient’s stable blood pressure and limited cardiovascular surgical staffing. No adjustment of SB tube pressure was performed during the ICU stay. No overt signs of recurrent gastrointestinal bleeding were observed during the ICU stay. However, ∼3 h after ICU admission, the intra-abdominal pressure, measured by intravesical pressure, increased to 26 mm Hg, exceeding the 20-mm Hg threshold for abdominal compartment syndrome (ACS), and was accompanied by hypotension. Follow-up CT revealed a considerably enlarged gastric hematoma and pneumoperitoneum, indicating ACS and gastric rupture due to intragastric hematoma expansion. TEVAR was initially performed using a thoracic stent graft (TGM262615J; WL Gore & Associates, Flagstaff, AZ, USA) deployed in zone 4, and the absence of an endoleak was confirmed (Fig. 1C). Subsequently, exploratory laparotomy was performed for abdominal decompression and exploration of the suspected perforation. The stomach was markedly distended. A small incision was made in the stomach, and the hematoma was evacuated. Examination of the other organs revealed no findings that could account for the increased intra-abdominal pressure; therefore, ACS secondary to acute gastric distension was diagnosed. The cause of the pneumoperitoneum was not identified during the initial surgery. Given the patient’s hemodynamic instability, we performed open abdominal management using a negative-pressure wound therapy system (ABTHERA; 3M, St. Paul, MN, USA), with a second-look surgery and abdominal closure scheduled the following day. Second-look surgery revealed a 2-cm perforation in the anterior wall of the gastric body. Because prolonged postoperative fasting was anticipated, an enterostomy was performed. The patient was successfully extubated 2 days after the initial surgery and was transferred from the ICU to the general ward on postoperative day 4, with a total ICU stay of 5 days. EGD performed 11 days after the initial surgery revealed exposure of the stent-graft within the esophageal lumen (Fig. 1D), suggesting a high risk of stent-graft infection. No cancer recurrences were observed. Thirteen days after the initial surgery, the patient developed a fever and persistent bacteremia. Stent-graft infection was suspected based on the clinical course and EGD findings. Definitive surgery was considered. However, the patient declined further invasive treatment, and a decision was made to pursue the best supportive care. The patient was discharged on day 29 of hospitalization. The patient died 80 days after the initial surgery. No recurrent gastrointestinal bleeding was observed after discharge, and the infection was controlled with oral antibiotics. Written informed consent for publication of the patient’s clinical information and images was obtained from the patient.

**Figure 1:**
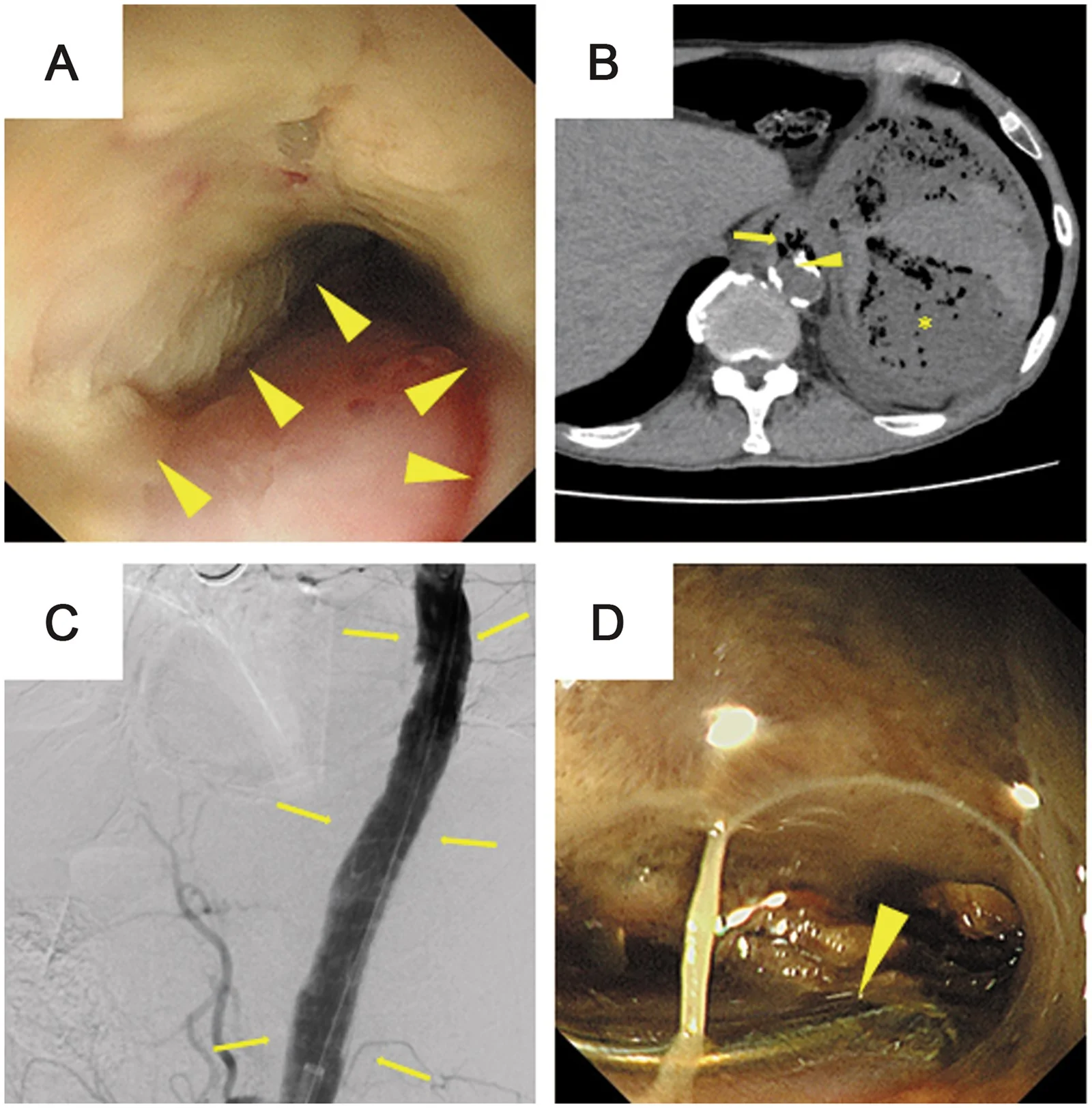
Endoscopic and computed tomographic findings of the aortoesophageal fistula and its treatment. (A) A circumferential longitudinal ulcer extending 40 cm from the incisors to the esophagogastric junction (EGJ) is noted, with no evidence of perforation. Arrowheads indicate the ulcer margins. (B) Contrast-enhanced computed tomography reveals fistulous communication between the EGJ and the anterior wall of the descending thoracic aorta (arrow), an aortic wall defect (arrowhead), and a large intragastric hematoma (asterisk). (C) The stent graft is successfully deployed in zone 4 of the descending thoracic aorta, and the absence of an endoleak (type I/III) is confirmed (arrow). (D) A deep ulcer is identified ∼1 cm proximal to the EGJ, with partial exposure of the stent graft at the ulcer base (arrowhead).

## Discussion

AEF is a fatal condition requiring surgical intervention. Open repair (aortic reconstruction with resection of the involved esophagus) remains the only definitive treatment for AEF. However, operative mortality remains high despite recent advances in surgery [1]. TEVAR has recently emerged as a minimally invasive and rapid treatment option for AEF [1, 2]. However, prompt TEVAR is often challenging in resource-limited settings. The use of esophageal balloon tamponade as a bridge therapy for TEVAR has been previously reported. Negoto *et al.* [3] described successful bridging with an SB tube, whereas Joseph *et al.* [4] used a 26-Fr Foley catheter as an alternative tamponade device before TEVAR. In our case, ACS and gastric perforation developed after SB tube placement, secondary to progressive gastric distension. We speculate that incomplete hemostasis contributed to gradual expansion of the intragastric hematoma, whereas direct compression of the gastric wall by the SB tube may have also contributed to gastric distension and subsequent perforation. Therefore, the SB tube should only be regarded as a temporary bridge. Although radiation-induced AEF has been reported in rare cases [5], to the best of our knowledge, AEF following heavy-ion radiotherapy has not been reported. In our case, the lesion was located near the esophagogastric junction, where exposure to refluxed gastric contents and acid may have contributed to the progression of the radiation-induced ulcer.

## Conclusions

The initial management of AEF is challenging, and TEVAR may not be available at all institutions. In such settings, the insertion of an SB tube may serve as a temporary bridging strategy until TEVAR can be performed.

## Authors’ contributions

R.S. performed the exploratory laparotomy, provided postoperative care, and drafted the manuscript. K.H. and Y.K. performed TEVAR, and K.N. contributed to the insertion of the SB tube. H.K. supervised the study and critically revised the manuscript. All authors read and approved the final manuscript.
